# Burnout pathway differences across dual-track residency training: a network analysis

**DOI:** 10.3389/fpubh.2026.1921620

**Published:** 2026-10-13

**Authors:** Yuduo Guo, Xiaonan Meng, Sichang Chen, Xiaoning Lan, Haoyuan Guo, Mingchu Li

**Affiliations:** 1 Department of Neurosurgery, Xuanwu Hospital, China International Neuroscience Institute (China-INI), Capital Medical University, Beijing, China; 2 Department of Acupuncture and Moxibustion, Beijing Huguosi TCM Hospital, Affiliated with Beijing University of Chinese Medicine, Beijing, China; 3 Department of Neurosurgery, Sanbo Hospital, Capital Medical University, Beijing, China

**Keywords:** bridge centrality, burnout, China, medical education, network analysis, residency training

## Abstract

**Background:**

China’s standardized residency training operates under a dual-track system: one track integrates clinical training with concurrent degree pursuit (student group), while the other is purely vocational without degree conferral (non-student group). Prior burnout research has largely relied on aggregate total or subscale scores, which cannot detect qualitative differences in symptom-to-symptom connectivity even when mean burnout severity is similar. Despite identical clinical rotations and competency requirements, it remains unclear whether these structurally distinct training pathways are associated with different burnout symptom-connectivity patterns, with direct implications for track-specific educational support.

**Methods:**

We conducted a secondary analysis of data from Fan et al.’s previously published cross-sectional survey of 636 residents from a Chinese tertiary teaching hospital. Burnout was measured using the 22-item Maslach Burnout Inventory-Human Services Survey (MBI-HSS). Networks were estimated using Graphical Lasso, and bridge centrality was computed to identify symptoms that most strongly connect burnout dimensions. Analyses were stratified by gender and specialty, and supplemented by logistic regression and bootstrap resampling.

**Results:**

Non-students showed higher emotional exhaustion and lower personal accomplishment scores than students, whereas depersonalization scores did not differ significantly between groups. Burnout prevalence was higher among non-students (50.2% vs. 42.4%), but this difference was not statistically significant. In both groups, DP2 (“I don’t really care what happens to patients”) was the dominant bridge node. However, PA4 (“I don’t easily understand how my patients feel”) entered the top five bridge items only in non-students. Three of four subgroups followed the DP2 pathway, whereas non-student males showed a distinct pattern with PA4 as the most prominent feature (BS = 1.445). Logistic regression found no significant association between training identity and burnout status after adjustment (OR = 1.07; ΔAUC = +0.002, *p* = 0.68), whereas network analyses revealed distinct symptom-connectivity patterns.

**Conclusion:**

These findings suggest that burnout prevention should account for training structure and institutional context. Rather than uniform wellness programs, medical education should offer track-specific support, particularly for residents whose burnout is characterized by empathy-related frustration rather than depersonalization.

## Introduction

China’s Standardized Resident Training (SRT) program, instituted nationwide in 2014, represents a monumental reform aimed at homogenizing the quality of clinical postgraduate medical education (1). However, this macro-level policy framework has structured a unique “dual-track” training modality that yields a profound institutional dichotomy among residents: the student track and the non-student track. Residents on the student track are concurrently enrolled in academic degree programs (Professional Master’s or Doctoral candidates) and are integrated into university-affiliated support systems, mentorship networks, and clear career-escalation pathways. Conversely, non-student residents, comprising social recruits and institutional commission trainees, undergo identical clinical rotations, undertake equivalent shifting workloads, and face the same standardized graduation examinations, yet they lack the formal academic identity and the protective institutional scaffolding afforded to their student-track peers. Emerging evidence suggests that professional identity among Chinese SRT trainees is significantly associated with burnout and psychological distress (2), and that a sense of school belonging may buffer against burnout (3), pointing to the importance of the institutional affiliation dimension of training. While this administrative cleavage is well-recognized, whether and how this structural asymmetry relates to differences in the underlying relational patterns of psychological distress between these two parallel resident cohorts remains critically under-explored.

The pervasive escalation of resident burnout within this high-stakes environment has become a significant concern in medical education, with severe implications for trainee well-being and clinical learning environments (4–7). A substantial body of literature has dedicated itself to mapping the risk factors of resident burnout, consistently identifying female gender and heavy work demands, including prolonged working hours, as primary correlates (8). Nevertheless, the vast majority of existing empirical studies typically operationalize burnout through aggregated dimensional scores derived from instruments like the Maslach Burnout Inventory (MBI), such as emotional exhaustion (EE), depersonalization (DP), and reduced personal accomplishment (PA). By relying on traditional latent variable frameworks, these studies implicitly focus on aggregated severity, assuming that the syndromic structure of burnout remains invariant across divergent groups. Consequently, they leave a critical conceptual gap: whether distinct training identities are associated with different structural patterns in how individual burnout symptoms interconnect.

The Job Demands-Resources (JD-R) model provides a useful theoretical lens to interpret these potential structural variations (9). The JD-R framework conceptualizes workplace well-being as a balance between job demands and job resources, involving parallel health-impairment and motivational pathways (10). Applied to China’s dual-track SRT system, while both student and non-student residents are subjected to identical clinical demands, their organizational resource pools are asymmetrical. For student residents, the concurrent pursuit of an academic degree may serve as a salient institutional resource that supports professional identity development (2, 11). For non-student residents, the absence of this specific institutional anchor represents a relative resource limitation (3). Viewed through this framework, such asymmetric resource distribution may imply potential differences within the dual-track system, manifesting not merely as variations in overall burnout scores, but in the specific relational networks among exhaustion, detachment, and reduced personal accomplishment across the two cohorts. Because student and non-student residents share identical clinical environments, rotation schedules, and assessment standards, the dual-track structure provides a quasi-experimental contrast in which between-group differences in symptom-network organization can be attributed to training identity and its associated institutional resources rather than to environmental confounders.

To capture these intricate, symptom-level relationships, network psychometrics offers a valuable descriptive tool. Rather than treating burnout as an unobservable common cause reflected uniformly in summary scores, network analysis conceptualizes psychological syndromes as systems of interacting components (12–14). Within this relational framework, “bridge centrality” serves as a structural metric to identify specific nodes that bridge distinct dimensions, highlighting key informational connections within the network topology (13). Recent network studies have consistently identified depersonalization symptoms as central bridge nodes connecting burnout dimensions across diverse occupational samples (12, 15), and have demonstrated that burnout network configurations can vary depending on demographic and institutional contexts (12, 16, 17). Large-scale network analyses among Chinese medical trainees have found that emotional exhaustion and cynicism frequently serve as bridge symptoms linking burnout with depression, anxiety, and dropout intention (18, 19). Network analysis has been increasingly utilized to explore psychological distress among medical trainees and faculty. However, its application to comparing the fine-grained structural features of burnout networks between distinct training tracks within a unified residency system has not, to our knowledge, been previously examined.

To address these gaps, this study examines whether the dual-track residency training structure is associated with different burnout symptom networks among student and non-student residents in China (20). Using network analysis and bridge centrality approaches, we aim to identify key symptoms and potential pathways underlying burnout across different training pathways, thereby providing insights for targeted educational support strategies. Furthermore, gender and specialty may moderate burnout-symptom networks: gender is linked to distinct distress-expression norms, with males socialized toward reduced emotional disclosure and help-seeking (21), whereas surgical and non-surgical specialties differ in patient-interaction intensity, workload patterns, and professional-identity experiences (8). Because these variables are deeply intertwined with professional socialization in medical education (8, 17), we conducted exploratory gender-stratified and specialty-stratified network comparisons to map potential sub-group variations. To isolate the unique structural features associated with training identity, we implemented covariate-adjusted regression and sensitivity analyses to account for potential confounding variations in baseline workloads. We hypothesized that training identities would exhibit distinct relational network structures of burnout, with potential topological and bridge node variations spanning across different demographic and specialty subgroups.

## Methods

### Study design and participants

This is a secondary analysis of data originally collected by Fan et al. (20), who conducted a cross-sectional survey of resident physicians at West China Hospital, Sichuan University, from February to June 2024. The original study was approved by the Ethics Review Committee of West China Hospital (2024–183). Participants provided informed consent by completing the electronic questionnaire. This secondary analysis used only de-identified, publicly available data; in accordance with Article 32 of the Ethical Review Measures for Life Science and Medical Research Involving Humans (National Health Commission of China, 2023), no additional ethical approval was required. Of 820 invited residents, 660 responded (80% response rate), and after excluding 24 invalid questionnaires (due to duplicate responses, completion time < 120 s, or inconsistent responses), 636 valid questionnaires were included. This secondary analysis included all 636 participants from the original study without additional exclusion criteria. The original study examined the relationship between person–vocation fit and burnout among resident physicians, using descriptive statistics, between-group comparisons, and multivariable logistic regression to identify burnout-associated factors. The present secondary analysis added analyses not performed in the original study: Gaussian graphical network estimation (Graphical Lasso), bridge centrality computation, network comparison tests, bootstrap stability procedures, and network analyses stratified by gender and specialty.

### Measures

#### Burnout

Burnout was assessed using the Chinese version of the Maslach Burnout Inventory-Human Services Survey (MBI-HSS) (22), which contains 22 items across three dimensions: Emotional Exhaustion (EE; 9 items), Depersonalization (DP; 5 items), and Personal Accomplishment (PA; 8 items). Each item is scored on a 7-point Likert scale from 0 (“never”) to 6 (“every day”), yielding a total score range of 0–54 for EE, 0–30 for DP, and 0–48 for PA. Higher scores on EE and DP indicate greater burnout, while higher scores on PA indicate greater personal accomplishment. In this sample, Cronbach’s alpha coefficients were 0.90 for EE, 0.83 for DP, and 0.85 for PA, indicating satisfactory internal consistency.

#### Training identity

Participants self-reported their training identity as one of four categories: social trainee, commission trainee from an outside hospital, commission trainee from the same hospital system, or professional master’s student. In line with the structural distinction in China’s residency training system, we recoded these into two groups reflecting whether the resident concurrently holds a degree-seeking status during training (student group: professional master’s and doctoral candidates, *n* = 337) or does not (non-student group: social and commission trainees, *n* = 299).

### Statistical analysis

#### Descriptive statistics

Demographic characteristics and MBI dimension scores were compared between the two groups using independent t-tests for continuous variables and chi-square tests for categorical variables. Burnout was defined as the concurrent presence of high EE (≥27), high DP (≥10), and low PA (≤33) following established Chinese cutoffs (22).

#### Network analysis

Gaussian graphical models were estimated using the Graphical Lasso algorithm with the Extended Bayesian Information Criterion (EBIC) for model selection (EBIC hyperparameter gamma = 0.5) (23, 24). Nodes represented the 22 MBI items, and edges represented partial correlations between two items after controlling for all other items. Network density was computed as the proportion of non-zero edges among all possible edges. Mean predictability (the proportion of variance in each node explained by all other nodes) was calculated using linear regression to evaluate the local interpretability of the estimated edges (25). To identify symptoms that most strongly connect different burnout dimensions, we computed bridge strength (BS) for each node, defined as the sum of absolute edge weights connecting that node to nodes in other dimensions (EE-DP, EE-PA, DP-PA) (13). The Network Comparison Test (NCT) was conducted to formally assess differences in network structure between groups, with 1,000 permutations (26).

#### Stability analysis

To evaluate the stability of bridge centrality estimates, two bootstrap procedures were employed. First, a case-dropping bootstrap was performed following the procedure described by Epskamp et al. (27). Cases were randomly removed from each group at increasing proportions (5 to 75% in 10% increments), and at each level the network was re-estimated and bridge strength recomputed. The correlation between the full-sample and subsample bridge strength vectors was calculated using Spearman’s rho. The correlation stability coefficient (CS-coefficient) was defined as the maximum proportion of cases that could be dropped while maintaining a correlation of at least 0.70 with the full-sample estimates. CS-coefficients ≥0.50 were considered indicative of adequate stability. Second, to obtain uncertainty intervals for individual bridge strength estimates, non-parametric bootstrap resampling was performed (500 iterations, 80% sampling fraction per iteration) for each group, from which node-wise 95% percentile confidence intervals were derived.

#### Subgroup and exploratory analyses

To examine whether network patterns varied by gender and specialty, we stratified the sample into four subgroups by training identity and gender (student-male, student-female, non-student-male, and non-student-female) and further by specialty (surgical vs. non-surgical) within each stratum. Networks were estimated and bridge centrality computed separately for each subgroup using the same Graphical Lasso parameters. In addition, Pearson correlations between workload indicators (working hours, sleep duration) and MBI item scores were computed separately for each group to explore potential mechanisms underlying network differences.

#### Logistic regression

To examine whether training identity predicted burnout status independently of demographic and workload confounders, we conducted hierarchical logistic regression. Model 1 included demographic variables (gender, age, specialty); Model 2 added workload variables (working hours, sleep duration, night shifts); Model 3 added training identity (student vs. non-student). Model fit was evaluated using area under the receiver operating characteristic curve (AUC).

All network analyses were conducted in Python 3.12 (scikit-learn for Graphical Lasso, NetworkX for graph operations, with custom functions for bridge centrality computation). Logistic regression was performed using scikit-learn. Bootstrap analyses and subgroup network estimations used parallelized resampling routines built on NumPy.

## Results

### Participant characteristics

A total of 636 residents were included, comprising 337 in the student group (professional master’s and doctoral candidates) and 299 in the non-student group (social and commission trainees). The demographic and clinical characteristics of participants are summarized in Table 1. Compared with non-students, students were younger and had higher proportions of male residents and of surgical specialties. The two groups showed comparable PGY distributions, while differences were observed in working hours and sleep duration (Table 1).

**Table 1:** Demographic and clinical characteristics by training group.

Variable	Student (*n* = 337)	Non-student (*n* = 299)	*p*-value
Age (years)	25.2 ± 2.2	25.9 ± 2.2	<0.001
Gender			
Male	218 (64.7%)	116 (38.8%)	
Female	119 (35.3%)	183 (61.2%)	<0.001
Specialty			
Surgical	250 (74.2%)	53 (17.7%)	
Non-surgical	87 (25.8%)	246 (82.3%)	<0.001
PGY			
PGY-1	135 (40.1%)	104 (34.8%)	
PGY-2	104 (30.9%)	94 (31.4%)	
PGY-3	98 (29.1%)	101 (33.8%)	0.315
Working hours (h/day)	10.7 ± 1.2	10.9 ± 1.2	0.024
Sleep duration (h/day)	5.6 ± 0.8	5.9 ± 0.8	<0.001
Night shifts (per month)	4.3 ± 1.6	4.0 ± 1.8	0.045
Burnout, *n* (%)	143 (42.4%)	150 (50.2%)	0.061
MBI-EE	28.9 ± 11.8	32.3 ± 11.7	<0.001
MBI-DP	12.4 ± 7.2	13.5 ± 7.6	0.068
MBI-PA	29.6 ± 9.3	27.2 ± 9.8	0.001

Data are presented as mean ± SD or *n* (%).

The overall burnout prevalence was 46.1% among all participants. Although the non-students showed a higher prevalence of burnout than students, the difference was not statistically significant. Regarding MBI subscales, non-students exhibited higher emotional exhaustion and lower personal accomplishment scores, whereas depersonalization scores did not differ significantly between groups (Table 1). These results are descriptive baseline contrasts (Table 1); the study’s primary focus is not these mean-level differences but the subsequent symptom-network comparisons.

### Network structure by training identity

Partial correlation networks were conditionally estimated based on the same 22-item MBI structure for both cohorts (Figure 1A). The student network exhibited 137 non-zero edges (density = 0.593), whereas the non-student network comprised 131 non-zero edges (density = 0.567). Notably, the EE6-DP2 edge, connecting the peak exhaustion item (EE6 “I feel like I’m at the end of my rope”) with the core depersonalization item (“I don’t really care what happens to patients”), was the strongest cross-dimension connection in both networks (non-students: *r* = 0.432; students: *r* = 0.264, Figure 1A), with this cross-cohort divergence formally evaluated below.

**Figure 1:**
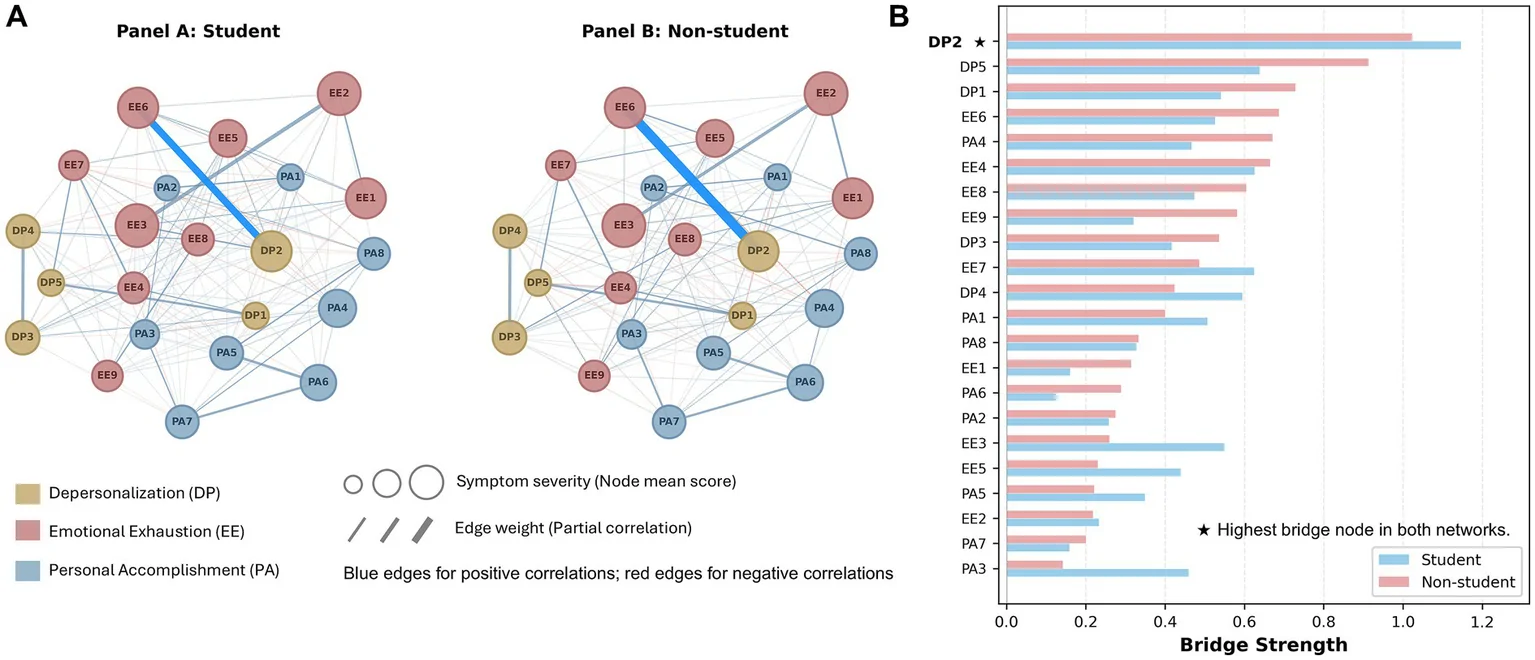
Network structures and bridge centrality profiles by training identity. **(A)** Regularized partial correlation networks for students (left) and non-students (right). Nodes represent the 22 MBI items color-coded by burnout dimension. Blue edges denote positive correlations, with thickness indicating weight magnitude. The prominent blue line highlights the primary cross-dimension connection (EE6–DP2). **(B)** Graphical comparison of item-specific bridge strength values between cohorts. DP2 serves as the dominant bridge node across both groups, while PA4 exhibits a marked rank-order disruption.

Centrality indices further revealed that DP2 emerged as the dominant bridge node in both networks, exhibiting higher bridge strength (BS) value in students than in non-students (BS = 1.147 vs. 1.024; Figure 1B; Table 2). Meanwhile, PA4 (“I don’t easily understand how my patients feel”) ranked among the top five bridge nodes exclusively in the non-student group (BS = 0.671; Rank 5), but did not enter the top 10 bridge nodes in the student group (BS = 0.467; Figure 1B). The top 10 bridge nodes for each group are presented in Table 2.

**Table 2:** Top ten bridge nodes by training group.

Rank	Node	BS (student)	Node	BS (non-student)
1	**DP2**	1.147	**DP2**	1.024
2	DP5	0.639	DP5	0.913
3	EE4	0.626	DP1	0.729
4	EE7	0.625	EE6	0.688
5	DP4	0.595	**PA4**	0.671
6	EE3	0.549	EE4	0.665
7	DP1	0.541	EE8	0.605
8	EE6	0.526	EE9	0.582
9	PA1	0.507	DP3	0.537
10	EE8	0.474	EE7	0.486

BS, Bridge strength. Nodes are ranked by bridge strength within each group. Bold values indicate the key bridge nodes discussed in the text: DP2 was the dominant bridge node in both groups, and PA4 entered the top five bridge nodes exclusively in the non-student group.

Network Comparison Test indicated high similarity in network structure (r = 0.844) and bridge strength distribution (*r* = 0.721), with several edge-level differences reaching nominal significance (*p* < 0.05), including EE6-DP2 (*z* = −2.39, *p* = 0.017; Supplementary Table S1).

### Gender and specialty stratified analysis

Prompted by the localized network differences identified above, the cohorts were further cross-stratified by gender into four subgroups: student-male (*n* = 218), student-female (*n* = 119), non-student-male (*n* = 116), and non-student-female (*n* = 183). While DP2 remained the highest bridge node in three subgroups (student-male, student-female, and non-student-female), PA4 emerged as the leading bridge node exclusively in the non-student-male subgroup, exhibiting the highest bridge strength value observed across all subgroups (BS = 1.445; Figure 2).

**Figure 2:**
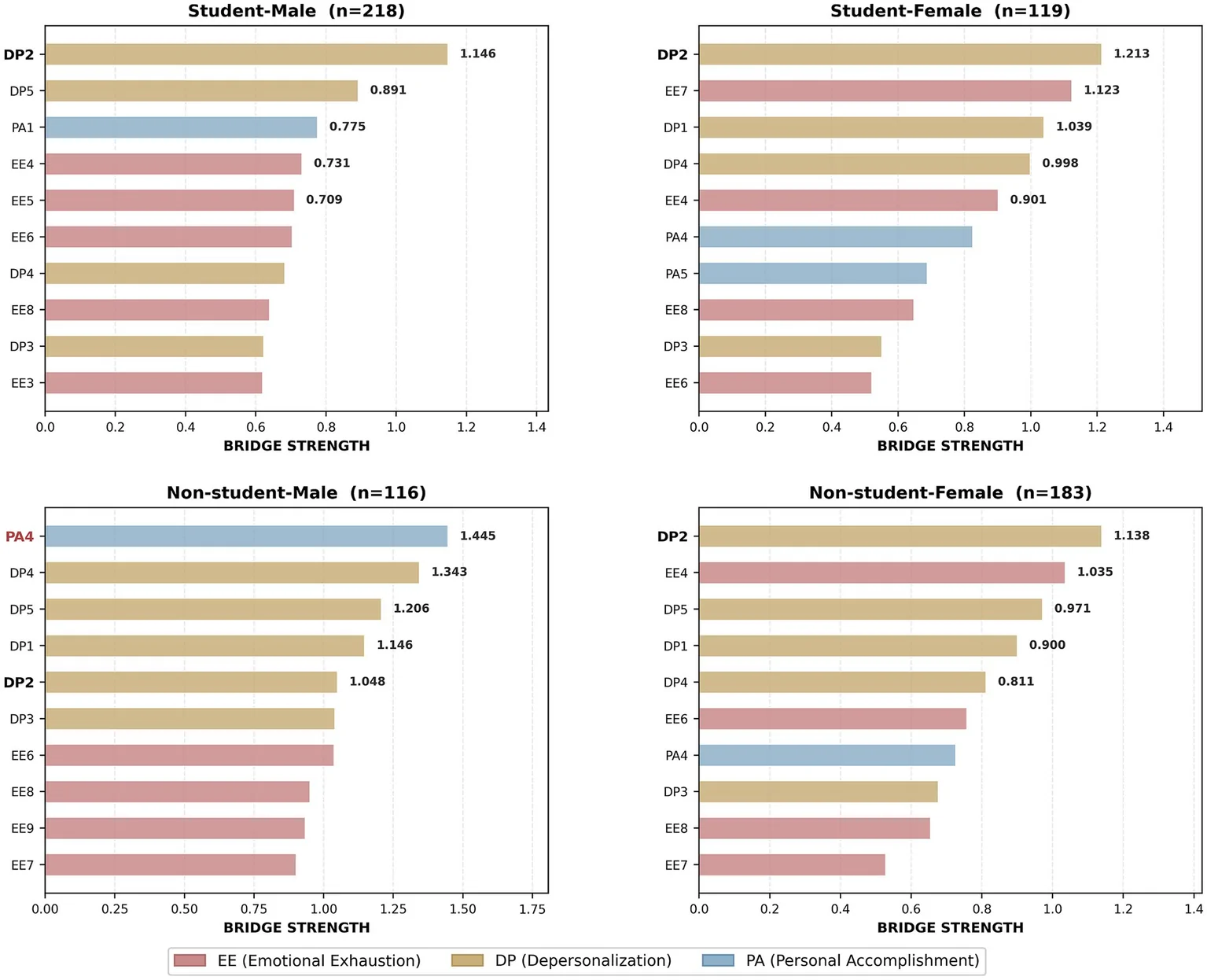
Bridge strength centrality profiles across the four gender- and identity-stratified subgroups.

To determine whether this PA4 dominance in non-student males was attributable to specialty composition, specialty-stratified analyses were performed. PA4 ranked first among non-student-male non-surgical residents (BS = 1.644), fifth in the non-student-male surgical subgroup (BS = 1.709). In contrast, this specialty-specific pattern was not observed in student-male residents, where PA4 failed to enter the top 10 bridge items in either specialty subgroup (Supplementary Table S2).

The panels display the bridge strength of burnout networks for student-male (*n* = 218), student-female (*n* = 119), non-student-male (*n* = 116), and non-student-female (*n* = 183) cohorts.

### Stability of bridge centrality estimates

To verify the robustness and reproducibility of the identified bridge strength profiles, case-dropping bootstrap resampling was performed (Figure 3A). The correlation stability (*CS*) coefficient (*cor* = 0.7) reached 0.55 for the student network and 0.65 for the non-student network, both exceeding the recommended threshold of 0.50, indicating adequate stability. Furthermore, bootstrap resampling (500 iterations, 80% sub-sampling) confirmed that PA4 consistently ranked among the top five bridge items in the non-student group (mean BS = 0.831; 95% CI 0.587–1.112) but did not exhibit comparable centrality ranks in the student group (mean BS = 0.650; 95% CI 0.428–0.920; Figure 3B). DP2 remained the top-ranked bridge node in both networks across all iterations.

**Figure 3:**
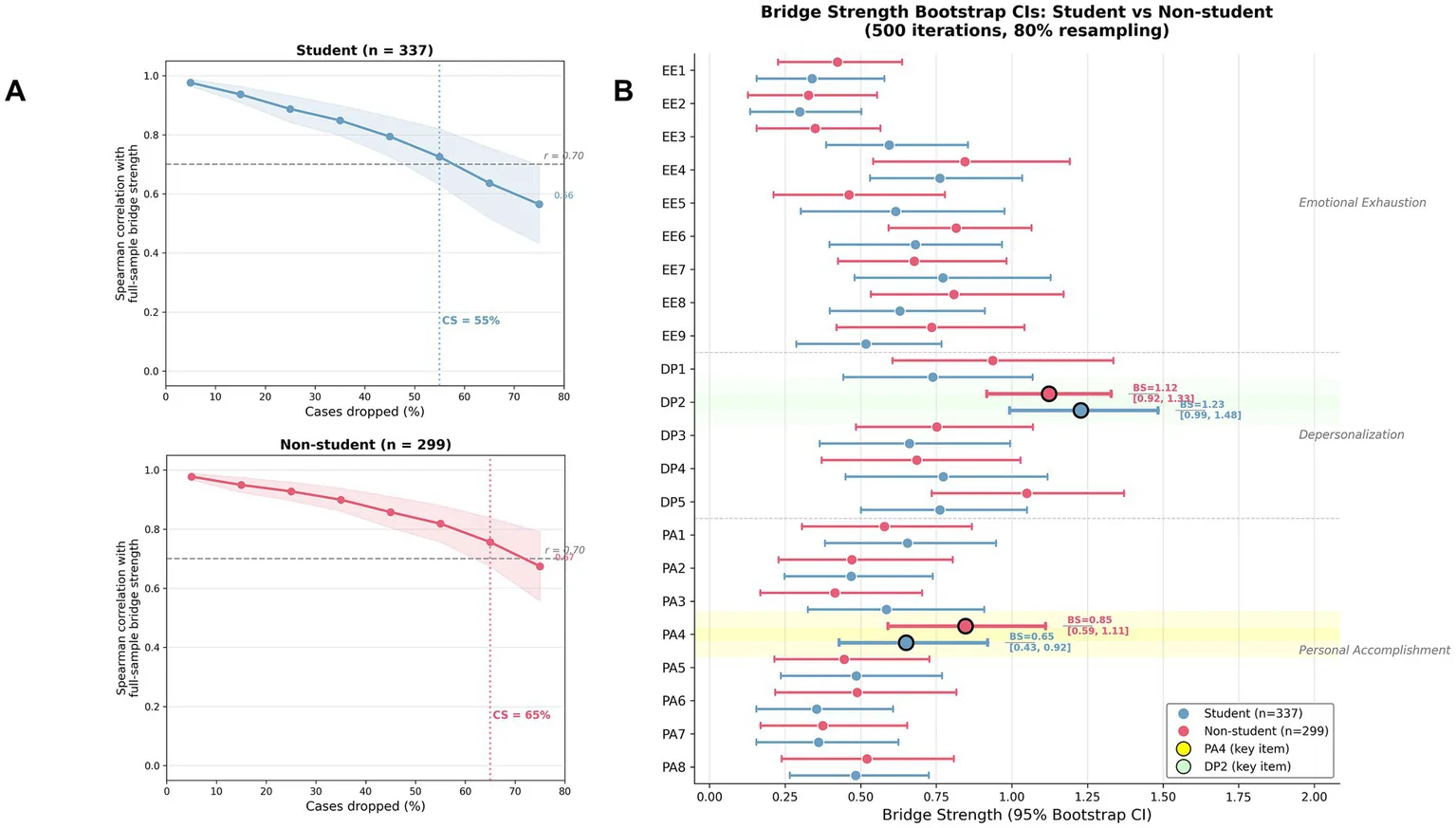
Robustness and stability of bridge strength centrality. **(A)** Case-dropping bootstrap resampling curves for students (*n* = 337) and non-students (*n* = 299). Dashed lines indicate the correlation stability (*CS*) coefficient at the *r* = 0.70 threshold. **(B)** Bootstrapped 95% confidence intervals (CIs) of bridge strength centrality (500 iterations; 80% sub-sampling). Highlighted bands indicate key bridge items: DP2 (green) and PA4 (yellow).

### Supplementary analyses

Logistic regression, after adjusting for age, gender, specialty, working hours, sleep duration, and night shifts, showed that training identity was not significantly associated with burnout status (OR = 1.07, 95% CI 0.74–1.55, *p* = 0.68; ΔAUC = +0.002; Supplementary Table S3). This null finding indicates that training identity did not predict burnout case/non-case status after covariate adjustment, even though the network analyses demonstrated qualitative differences in symptom connectivity patterns between the two groups. Exploratory correlational analyses showed stronger correlations between working hours and EE6 in non-students (*r* = 0.500 vs. 0.279) and stronger negative correlations between working hours and PA4 in non-students (*r* = −0.323 vs. −0.146).

In summary, the estimated burnout networks demonstrated high global structural alignment and adequate parameter stability, with DP2 acting as the primary bridge node across the majority of strata, except for the non-student-male non-surgical subgroup where PA4 exhibited the highest centrality.

## Discussion

To our knowledge, this is the first network analysis to compare burnout network structures between student and non-student residents in China’s dual-track training system. We found that the overall network architecture was similar between the two groups, with DP2 (“I don’t really care what happens to patients”) as the strongest bridge node in both. However, an important difference appeared when we considered training identity together with gender. In non-student male residents, empathy-related frustration (PA4, “I don’t easily understand how my patients feel”) replaced DP2 as the strongest bridge node. This pattern could not be explained by demographic confounders, workload, or specialty composition. These findings indicate that the structural asymmetry in China’s dual-track training system is associated with qualitative differences in burnout patterns, not just differences in severity.

### The universal engine of resident depersonalization: DP2 as the core bridge node

The central role of DP2 as the primary bridge node in both groups is consistent with previous network studies showing that depersonalization symptoms serve as key connectors among burnout dimensions (12, 18). In the JD-R framework, depersonalization can be understood as a psychological distancing mechanism-a way to conserve resources when job demands exceed coping capacity (9, 28). In our data, the connection between EE6 (“I feel like I’m at the end of my rope”) and DP2 was particularly strong in non-student residents. This pattern fits the health-impairment pathway of the JD-R model: when institutional resources such as degree pursuit are absent, emotional exhaustion and psychological disengagement may become more tightly linked (9). Notably, the two groups had similar DP scores despite non-students reporting higher emotional exhaustion, suggesting that the key difference lies in how symptoms interconnect rather than in overall severity.

### The PA4 landscape shift in non-student male residents: potential interpretations and educational implications

The most distinctive finding from our network comparison is the localized topological shift surrounding PA4 in the non-student male subgroup. PA4 captures empathy-related frustration, a construct distinct from the emotional distancing measured by depersonalization items. In non-student males, PA4 replaced DP2 as the strongest bridge node, a pattern not explained by specialty composition or gender alone.

We interpret this finding through the lens of dual marginality, the intersection of structural identity and gender socialization within the medical hierarchy. As a possible contextual frame, this perspective suggests that non-student residents occupy an ambiguous professional position: they perform identical clinical work to their student peers but lack the formal academic affiliation that confers institutional recognition and a clear developmental trajectory. Professional identity formation in medical training relies heavily on institutional belonging and recognition within communities of practice (11, 29); when these pathways are structurally limited, empathy-related frustration may become a more salient dimension of burnout. Viewed through the JD-R framework, this structural resource deficit may be associated with heightened sensitivity to interpersonal aspects of clinical work, particularly the patient-physician relationship, precisely because professional identity cannot be affirmed through academic channels (9, 11). Among males, this pattern may be further shaped by gender socialization patterns that discourage help-seeking and emotional expression in distress (21); empathy frustration may represent a particularly salient aspect of burnout in this group. The exploratory workload interaction analysis is consistent with this interpretation: the correlation between working hours and PA4 was substantially stronger in non-students (*r* = −0.323 vs. –0.146), suggesting that identical workload burdens show a stronger association with empathy-related frustration in this group.

These findings carry direct educational implications. Conventional wellness interventions, such as generic stress management workshops or workload reduction, may be insufficient for non-student male residents, whose distress pattern uniquely involves empathy frustration rather than mere physical exhaustion. Targeted approaches, including Balint groups that focus on difficult patient encounters (30), structured empathy training programs (31), and peer support networks that normalize emotional challenges in clinical communication (32), may address this group’s specific vulnerability more effectively than generic burnout prevention strategies. Educational programs should consider whether the professional identity needs of non-student residents are adequately supported within existing training structures.

### Implications for other training systems

This study focused on China’s dual-track system, but the core idea, differences in training identity, may apply to other settings too. Many countries have similar structural divisions in residency training: academic versus community programs, MD versus MD-PhD tracks, or university-affiliated versus independent hospitals (11, 33). Residents in different tracks may receive different levels of institutional support and professional identity recognition, even when their clinical work is similar. The network analysis method we used offers a way to test whether such structural differences are linked to different burnout symptom patterns, not just different burnout levels. Future studies in other training systems could use similar methods to see whether our findings, especially the role of empathy-related frustration in groups with weaker institutional ties, replicate in other contexts.

### Methodological considerations and strengths

This study has several methodological strengths. The use of Graphical Lasso with EBIC model selection provided regularized network estimates that are robust to spurious edges (23). The stability of bridge centrality estimates was rigorously evaluated through multiple complementary approaches: case-dropping bootstrap procedures yielded correlation stability (CS) coefficients that exceeded the recommended threshold of 0.50 in both networks (27), non-parametric bootstrap with 500 iterations confirmed the reproducibility of PA4’s ranking in the non-student group (Figure 3B), the Network Comparison Test (NCT) confirmed that the global network structure was highly similar between the two primary cohorts (*r* = 0.844) (26), and bridge centrality computation followed established methodological recommendations (13). The complementarity of logistic regression and network analysis, with the former capturing null effects on burnout severity and the latter revealing qualitative pathway differences, underscores the value of multidimensional analytical strategies for understanding complex psychosocial phenomena.

### Limitations and future directions

Despite its strengths, several limitations must be acknowledged. First, the cross-sectional nature of the data precludes any definitive causal inferences regarding the directionality of the edges; although bridge nodes are conceptualized as potential intervention targets, longitudinal network designs (e.g., Ecological Momentary Assessment) are warranted to confirm the chronological evolution of these symptoms (34). Second, our sample was drawn from a single regional cohort of residents, which may limit the generalizability of the network topologies to different cultural or institutional contexts. The burnout prevalence in Chinese residency programs has been reported as approximately 70% in a multi-center study (35), and our findings should be validated in broader multi-regional samples. Third, although the overall sample size exceeded recommended minimums for network estimation (27), the three-way stratification into eight subgroups (training identity × gender × specialty) produced several underpowered strata (e.g., n = 19, n = 18), and these subgroup results should be interpreted cautiously. Larger multi-center samples would enable adequately powered comparisons across all strata. Fourth, the MBI-HSS measures perceived empathy-related frustration as a component of personal accomplishment rather than objective empathic ability; future studies incorporating behavioral measures of empathy would be valuable to disentangle these constructs.

## Conclusion

In conclusion, this study found that the overall burnout network structure was similar between student and non-student residents, but the specific patterns of symptom connections differed by training identity and gender. Non-student male residents showed a distinct pattern: empathy-related frustration, rather than depersonalization, was the strongest bridge symptom. These findings suggest that looking at individual symptom connections may be more informative than relying on total burnout scores alone. Medical education programs should consider track-specific wellness strategies rather than uniform interventions for all residents.

## Data Availability

Publicly available datasets were analyzed in this study. This data can be found at: https://doi.org/10.1371/journal.pone.0324707 (PLoS One Figshare Repository). The analysis code is available from the corresponding author upon reasonable request.
