# Supplementary material for: Burnout pathway differences across dual-track residency training: a network analysis
Source: Front Public Health. 2026 Oct 13;14:1921620. doi: 10.3389/fpubh.2026.1921620 (PMC13709981; doi:10.3389/fpubh.2026.1921620)
Supplement: Supplementary file 1 [file Table_1.DOCX]

**Supplementary Table S1. Network Comparison Test: edge-level differences between student and non-student networks**

*Fisher's z-test comparing edge weights. Only edges with nominally significant differences (p < 0.05) are shown. The key EE6–DP2 edge is highlighted in yellow.*

| **Edge** | **z** | **p** | **Student r** | **Non-student r** |
| --- | --- | --- | --- | --- |
| **EE6(EE)--DP2(DP)** | -2.39 | 0.017 | 0.264 | 0.432 |
| **EE4(EE)--DP5(DP)** | 2.19 | 0.028 | 0.045 | -0.129 |
| **EE2(EE)--EE3(EE)** | 2.18 | 0.029 | 0.646 | 0.533 |

*Global network structure similarity (partial correlation vectors): r = 0.844. Bridge strength distribution similarity: r = 0.721. These values indicate high overall structural similarity between the two networks despite nominal edge-level differences.*
