# Supplementary material for: Burnout pathway differences across dual-track residency training: a network analysis
Source: Front Public Health. 2026 Oct 13;14:1921620. doi: 10.3389/fpubh.2026.1921620 (PMC13709981; doi:10.3389/fpubh.2026.1921620)
Supplement: Supplementary file 2 [file Table_2.DOCX]

**Supplementary Table S2. Specialty-stratified bridge centrality results across eight subgroups**

*Only subgroups with n ≥ 30 are reported with full Top 10 bridge nodes. Subgroups with insufficient sample size are marked. BS = bridge strength. PA4 rows are highlighted in bold with yellow background.*

| **Subgroup** | **n** | **Rank** | **Node (Dimension)** | **Bridge Strength (BS)** |
| --- | --- | --- | --- | --- |
| Student-Male-Surgical | 199 | 1 | DP2(DP) | 1.157 |
|  |  | 2 | DP5(DP) | 0.96 |
|  |  | 3 | DP4(DP) | 0.795 |
|  |  | 4 | EE4(EE) | 0.774 |
|  |  | 5 | EE5(EE) | 0.744 |
|  |  | 6 | PA1(PA) | 0.731 |
|  |  | 7 | EE8(EE) | 0.685 |
|  |  | 8 | EE6(EE) | 0.659 |
|  |  | 9 | PA3(PA) | 0.584 |
|  |  | 10 | DP1(DP) | 0.569 |
| Student-Male-Non-surgical | 19 | — | Insufficient sample size | — |
| Student-Female-Surgical | 51 | 1 | DP1(DP) | 1.566 |
|  |  | 2 | DP4(DP) | 1.553 |
|  |  | 3 | DP3(DP) | 1.535 |
|  |  | 4 | DP2(DP) | 1.482 |
|  |  | 5 | EE7(EE) | 1.349 |
|  |  | 6 | DP5(DP) | 1.338 |
|  |  | **7** | **PA4(PA)** | **1.2** |
|  |  | 8 | EE6(EE) | 1.097 |
|  |  | 9 | PA3(PA) | 1.089 |
|  |  | 10 | EE1(EE) | 1.062 |
| Student-Female-Non-surgical | 68 | 1 | EE8(EE) | 1.1 |
|  |  | 2 | DP4(DP) | 1.09 |
|  |  | 3 | DP3(DP) | 1.055 |
|  |  | 4 | EE4(EE) | 1.027 |
|  |  | 5 | EE7(EE) | 1.013 |
|  |  | 6 | DP2(DP) | 1.011 |
|  |  | 7 | PA1(PA) | 0.928 |
|  |  | 8 | DP1(DP) | 0.898 |
|  |  | 9 | EE5(EE) | 0.793 |
|  |  | **10** | **PA4(PA)** | **0.686** |
| Non-student-Male-Surgical | 35 | 1 | DP2(DP) | 2.251 |
|  |  | 2 | DP3(DP) | 1.975 |
|  |  | 3 | DP5(DP) | 1.934 |
|  |  | 4 | DP4(DP) | 1.874 |
|  |  | **5** | **PA4(PA)** | **1.709** |
|  |  | 6 | PA2(PA) | 1.564 |
|  |  | 7 | EE8(EE) | 1.489 |
|  |  | 8 | PA8(PA) | 1.457 |
|  |  | 9 | EE6(EE) | 1.45 |
|  |  | 10 | PA5(PA) | 1.386 |
| Non-student-Male-Non-surgical | 81 | **1** | **PA4(PA)** | **1.644** |
|  |  | 2 | DP1(DP) | 1.529 |
|  |  | 3 | EE9(EE) | 1.318 |
|  |  | 4 | DP5(DP) | 1.273 |
|  |  | 5 | DP2(DP) | 1.27 |
|  |  | 6 | EE7(EE) | 1.254 |
|  |  | 7 | DP4(DP) | 1.24 |
|  |  | 8 | EE6(EE) | 1.21 |
|  |  | 9 | DP3(DP) | 1.17 |
|  |  | 10 | EE8(EE) | 1.163 |
| Non-student-Female-Surgical | 18 | — | Insufficient sample size | — |
| Non-student-Female-Non-surgical | 165 | 1 | DP2(DP) | 1.162 |
|  |  | 2 | EE4(EE) | 1.129 |
|  |  | 3 | DP5(DP) | 1.035 |
|  |  | 4 | DP1(DP) | 0.942 |
|  |  | 5 | DP4(DP) | 0.873 |
|  |  | 6 | EE6(EE) | 0.725 |
|  |  | 7 | DP3(DP) | 0.676 |
|  |  | **8** | **PA4(PA)** | **0.662** |
|  |  | 9 | EE8(EE) | 0.596 |
|  |  | 10 | EE7(EE) | 0.593 |

*Note: Bold rows with yellow background indicate PA4 ("I don't easily understand how my patients feel") — the key empathy-related bridge node.*
