# Supplementary material for: Burnout pathway differences across dual-track residency training: a network analysis
Source: Front Public Health. 2026 Oct 13;14:1921620. doi: 10.3389/fpubh.2026.1921620 (PMC13709981; doi:10.3389/fpubh.2026.1921620)
Supplement: Supplementary file 3 [file Table_3.DOCX]

**Supplementary Table S3. Logistic regression models predicting burnout status**

*Burnout was defined as high EE (≥27), high DP (≥10), and low PA (≤33). AUC = area under ROC curve.*

| **Model** | **Covariates** | **AUC** |
| --- | --- | --- |
| **Model 1: Demographics** | Age, gender, specialty | 0.603 |
| **Model 2: + Workload** | Model 1 + working hours, sleep duration, night shifts | 0.662 |
| **Model 3: + Training identity** | Model 2 + training identity (student vs. non-student) | 0.664 |
| **Model 4: + Identity × Gender** | Model 3 + identity-by-gender interaction | 0.664 |

**Key results (Model 3)**

Training identity coefficient: **0.069 (log-OR)**

Odds ratio (non-student vs. student): **1.07 (95% CI 0.74–1.55)**

p-value (identity): **0.68**

ΔAUC (Model 3 − Model 2): **+0.002**
